# A sustainable HPTLC approach for green assessment of Tyrosine to phenylalanine ratio in chronic kidney disease

**DOI:** 10.1038/s41598-024-79611-9

**Published:** 2024-11-17

**Authors:** Rania M. Kamel, Fatma A. M. Abdel-aal, Mostafa M. Osman, Fardous A. Mohamed, Abdel-Maaboud I. Mohamed

**Affiliations:** 1 Pharmaceutical Analytical Chemistry Department, Faculty of Pharmacy, Assiut University, Assiut, 71526 Egypt; 2 Urology Department, Faculty of Medicine, Al-Azhar University, Assiut, Egypt

**Keywords:** Biomarkers, Chronic kidney disease, HPTLC, Phenylalanine, Serum samples, Tyrosine, Biomarkers, Chemistry

## Abstract

The kidney is considered the main site for the net release of Tyrosine (Tyr) to the circulation through hydroxylation of Phenylalanine (Phe) using phenylalanine hydroxylase enzyme. In chronic kidney disease (CKD) patients the enzyme activity is impaired, therefore the serum concentration ratio of Tyr/Phe is reduced compared to healthy individuals. This motivates us to develop a cost effective, green, simple, sensitive, and selective HPTLC method to measure the levels of Tyr and Phe in serum samples. The analysis was carried out using acetonitrile: ethanol: 25% ammonia solution: ethyl acetate (6.5: 1.5: 1: 0.5) as a mobile phase. Rf values were 0.55 ± 0.05 for Phe and 0.39 ± 0.05 for Tyr. Densitometry scanning was performed using UV detector and dual wavelengths of 210 and 225 nm were obtained. A linear correlation was observed between the levels of Phe and Tyr, ranging from 50 to 700 ng band^− 1^ and 50 to 600 ng band^− 1^, respectively, under the optimum conditions. The method selectivity, linearity, precision, accuracy, and robustness were all confirmed in accordance with ICH recommendations. Calculations of the separation and resolution factors, number of theoretical plates, and height equivalent to theoretical plates prove to the chromatographic system accuracy and high separation efficiency. The developed method exhibits an acceptable eco-scale when measuring the method greenness using AGREE and GAPI softwares. It was applied for the determination of Phe and Tyr concentrations in human serum samples.

**Supplementary Information:**

The online version contains supplementary material available at 10.1038/s41598-024-79611-9.

## Introduction

Chronic kidney disease (CKD) is a global health burden with a high economic cost to health systems^1^. It is described as a condition where there are ongoing abnormalities in urine, structural abnormalities, or a decrease in the kidney’s ability to excrete waste that may indicate a reduction in functional nephrons^2^. Around 11% of the global population suffers from CKD^1^, which causes a gradual decline in kidney function. This can eventually result in end-stage renal disease, where dialysis or a kidney transplant becomes necessary. CKD in its early stages presents with minimal symptoms, making it difficult to detect until later stages when the risk of cardiovascular illness and death rises^3,4^. Consequently, it is crucial to pinpoint a predictive biomarker for CKD.

The maintenance of the body’s amino acid pools is largely dependent on the kidney, which accomplishes this task through various processes such as synthesis, degradation, filtration, reabsorption, and excretion via urine^5^. Among of those amino acids are Phenylalanine (Phe) and Tyrosine (Tyr).

Phe is an amino acid that humans cannot produce on their own, making it essential or necessary. On the other hand, Tyr is classified as a semi-essential or conditionally required amino acid since it can only be synthesized from Phe through hydroxylation using phenylalanine hydroxylase enzyme. That enzyme is present in three sites pancreas, liver, and kidney^6^. The kidney predominantly contributes to the supply of Tyr to the body’s circulatory system by converting Phe to Tyr^7,8^. Therefore, in case of CKD, there is a significant decrease in the Tyr release which leading to decrease in its serum levels as the phenylalanine hydroxylase enzyme activity is reduced^9^. Consequently, the Tyr/Phe concentration ratio is decreased compared to healthy individuals^6^. A shortage of Tyr may result in protein depletion as well as hindered production of aromatic amine modulators such as dopamine, norepinephrine, or epinephrine^5^. Therefore, Tyr can be considered an essential amino acid in patients with CKD.

Therefore, the ratio of serum Tyr to Phe levels could be used to evaluate the enzyme activity making it a potential biomarker for the early-stage detection of CKD.

The use of HPTLC technique in biomarker evaluation has numerous benefits such as low operational expenses, minimal sample clean up, and high sample throughput. Furthermore, this technique allows multiple samples to be processed simultaneously using small amounts of mobile phase solvents, resulting in reduced analysis time and cost compared to HPLC methods^10–13^.

Thus, our current study aimed to establish a validated HPTLC-densitometric method that is simple, sensitive, selective, green and economical for the determination of Tyr and Phe serum levels in both healthy individuals and CKD patients. The significance in the difference between Tyr/Phe concentration ratio in both studied groups was estimated through the determination of P-values and other statistical parameters such as t- test and *F*- test.

## Experimental

### Instrumentation

A Camag-HPTLC apparatus consisting of Camag TLC scanner III with visionCATS software version 3.1.21109.3 (Muttenz, Switzerland), that was adjusted for 20 mm/s scanning speed and 100 µL/step for data resolution. It was operated for the reflectance-absorbance mode using deuterium tungsten lamp. The apparatus also was equipped with linomat 5 for automatic application of sample. Sample syringe of 100 µL (Hamilton, Bonaduz, Switzerland) was used for the application of sample as a band under steam of nitrogen for the instant evaporation of solvents. HPTLC aluminium plates were coated with silica gel G60 F_254_ (20 × 10 cm). UV lamp (short wavelength 254 nm, Vilber Louranate 220 V 50 Hz, Marne-la-Vallee Cedex, France). TLC tank (standard type, 27.0 cm width x 26.5 cm height x 7.0 cm diameter, Sigma-Aldrich Co., USA) was used. Ultrasonic cleaner (Cole-parmer, Chicago, USA) and Sartorious handy balance- H51 (Hannover, Germany) were also used.

### Chemicals and reagents

Phe and Tyr standards were purchased from Sigma Aldrich (steinheim, Germany). HPLC-grade methanol was purchased from Sigma-Aldrich, Switzerland. Ammonia solution 25% and ethyl acetate (analytical grade) were purchased from El-Nasr pharmaceutical chemicals company, Egypt. Acetonitrile of HPLC grade was purchased from Fisher Scientific (U.K.) and ethanol (Merck, Darmstadt, Germany). All other chemicals and solvents used in this study were of analytical grade.

### Preparation of standard solutions

Standard stock solutions of both amino acids were prepared by dissolving an accurately weighed amount of 10 mg from both amino acids in water for Phe and 0.1 mol L^-1^ HCl for Tyr in 10 mL-volumetric flasks. Working solutions were prepared by dilution of the standard stock solution using methanol as a diluent to give working concentrations in the range of 10–140 µg mL^-1^ and 10–120 µg mL^-1^ for Phe and Tyr, respectively. All working solutions were prepared day by day from the stock solutions.

### Preparation of serum samples

This study was carried out in line with the principles of the Helsinki Declaration. Approval was granted by the Institutional Review Board of the Faculty of Medicine in Assiut University, Assiut, Egypt (approval No.04-2023-200151).

**Fig. 1:**
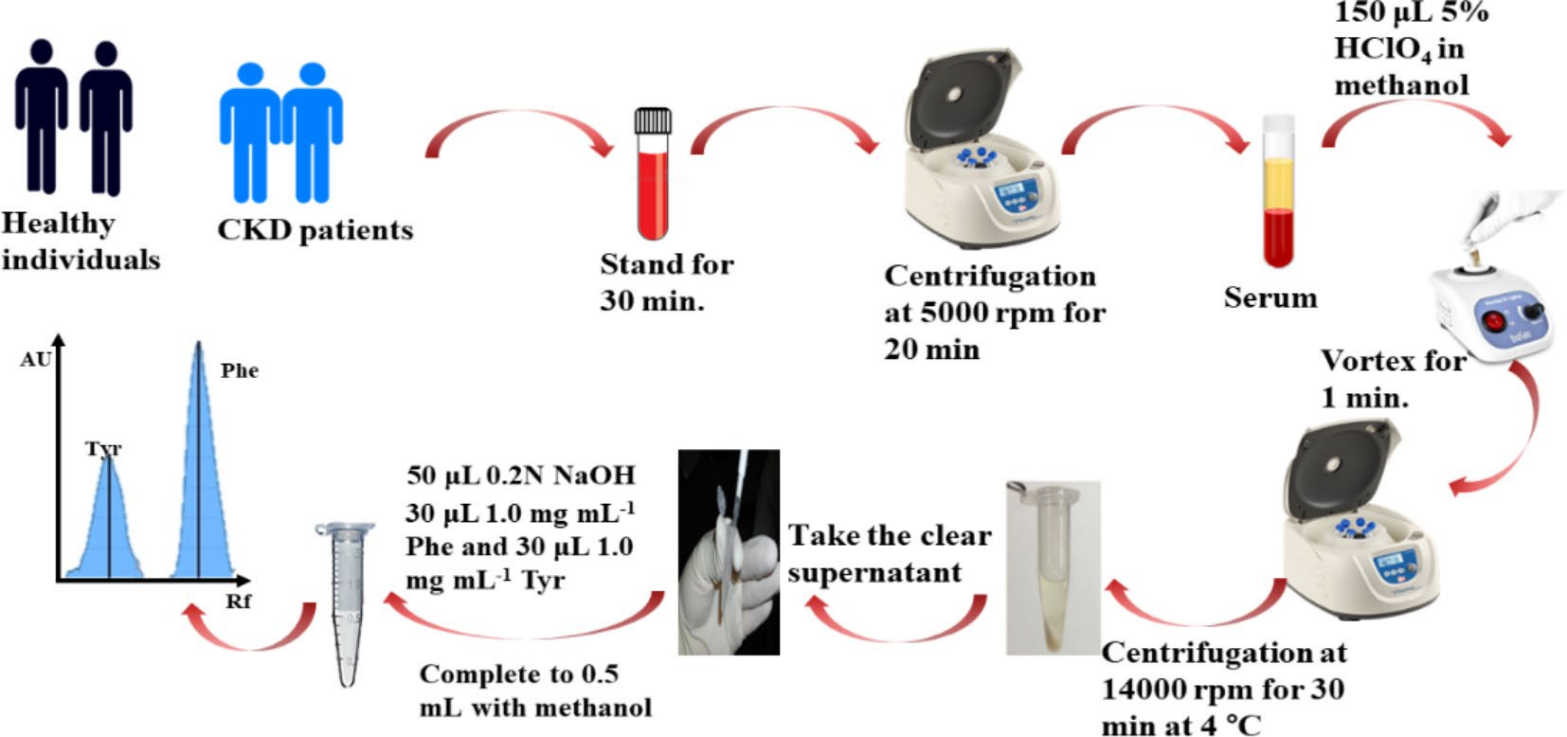
Procedure for the preparation of serum samples for Phe and Tyr determination by the developed method.

Blood samples were collected randomly from the veins of five healthy volunteers and ten chronic kidney disease patients (Samples were collected from the patients prior to the kidney dialysis session) in serum collecting tubes. Blood samples were left for 30 min to allow blood coagulation then centrifuge at 5000 rpm for 20 min. to separate the clot and obtain the serum samples. A volume of 150 µL of 5% perchloric acid in methanol were added to 300 µL of the serum sample then was centrifuged for 30 min at 14,000 rpm at 4 ºC to separate the precipitated protein. The supernatant was transferred to another clean Eppendorf tube (430 ± 5 µL) with the addition of 50 µL of 0.2 mol L^− 1^ NaOH to reduce the acidity of the supernatant. Standard addition method was carried out by adding 30 µL from standard Phe and Tyr stock concentrations (1.0 mg mL^− 1^). Finally, the volume was completed to 500 µL with methanol reaching to a final concentration of 60 µg mL^− 1^ for both amino acids. Figure 1 shows the procedure for the preparation of serum samples.

### Chromatographic conditions

TLC was carried out at silica gel G60 F_254_ layer with diameter of 0.2 mm and fixed in aluminium foil back plates. The methanol prewashed plates were cut into 20 × 10 cm pieces. Five microliters of standard working solutions or the prepared serum samples were applied to the plates by linomat 5 automated sample applicator with 6 mm band width and of 10 mm from the side edges and the bottom.

Acetonitrile: ethanol: 25% ammonia solution: ethyl acetate (6.5:1.5:1:0.5, v/v/v/v) was used as the mobile phase. The mobile phase was poured in the TLC tank contained filter paper to promote saturation ad covered with aluminium foil and the lid. It was kept for 15 min to saturate at room temperature (25 ± 2 ºC) then the plate was added to allow development for a migration distance of 80 mm.

After development, the plates were removed from the tank and dried in air for about ten min. The plates were scanned by Camage TLC scanner III with visionCATS software version 3.1.21109.3 at the absorption mode using dual wavelengths of 210 and 225 nm, respectively.

### Method validation

The validation of the proposed method was done according to ICH guidelines^14^. The studied validation parameters were selectivity, linearity and range, limit of detection and limit of quantitation, precision and accuracy, and robustness.

#### Selectivity

The separation factor (α) is a measure of the selectivity of the system to evaluate the system’s ability to separate two distinct compounds^15^. It is calculated by the following equation (Equ.1):

1 $$\alpha {\text{ }} = {\text{ }}[(1/Rf_{1} ){\text{ }} - {\text{ }}1\left] / \right[(1/Rf_{2} ){\text{ }} - {\text{ }}1]$$

(Where Rf_1_ and Rf_2_ correspond to the retardation factors of the analytes with higher and lower silica affinity, respectively)

To confirm a satisfactory separation, the value of the separation factor should exceed 1.0.

Moreover, the separation between two adjacent peaks in a chromatogram is measured by the resolution factor (Rs)^16^. It is important to evaluate the accuracy of the developed method for quantitative analysis. It is calculated by the following equation (Equ.2):

2 $$R_{S} = {\text{ }}\left( {z_{1} - z_{2} } \right)/\left( {0.5\left( {w_{1} + w_{2} } \right)} \right)$$

(Where **z**_**1**_ and **z**_**2**_ are the distance of separation between two adjacent peaks 1 and 2, **w**_**1**_ and **w**_**2**_ are the width of two adjacent peaks 1 and 2). A value greater than 1.0 for the Rs indicates satisfactory resolution between the two peaks.

Additionally, the number of theoretical plates (N) and height equivalent to theoretical plate (H or HETP) are a measure of the separation efficiency of a chromatographic system and the analyte spot broadening^16^. They could be calculated by the following equations (Equ.3 & 4):

3 $$N{\text{ }} = {\text{ }}\left( {16.\iota .z} \right)/w^{2}$$

4 $$N{\text{ }} = {\text{ }}\left( {\iota /H} \right)$$

(Where **w** is the width of the chromatographic spot or peak, $\iota$ and **z** are the migration distance of the mobile phase and the solute, respectively)

A chromatographic system is considered efficient if it has a small H value and a maximum N value, which can be achieved through a smaller particle size of the stationary phase, a low flow rate of the mobile phase, a less viscous mobile phase, and the presence of small solute molecules.

#### Linearity and range, limit of detection LOD and limit of quantitation LOQ 

To establish the linearity and range of the proposed method, six calibration mixtures of seven concentrations were analysed within the concentration range of 50–700 ng band^-1^ for Phe and 50–600 ng band^-1^ for Tyr.

Peak area and amino acid concentration values were handled by linear least square regression analysis to generate the calibration curve. In accordance with the outcomes of each regression equation for each amino acid, regression parameters were established, and the analysis of serum samples was also confirmed.

The values for LOD and LOQ were determined using the calibration curve results, as follows:

$$LOD{\text{ }} = {\text{ }}((3.3 \times S_{b} )/a)$$

$$LOQ{\text{ }} = {\text{ }}((10 \times S_{b} )/a)$$

Where the value of **S**_**b**_ refers to the standard deviation of the intercept, while **a** represents the slope of the calibration curve.

#### Precision and accuracy

The precision of the proposed method was evaluated by analyzing the repeatability (intra-day precision) and intermediate precision (inter-day precision) of the results. This was conducted at three different concentration levels (100, 300, 500 ng band^-1^ for both amino acids which represents low, medium, and high concentrations for the amino acids being studied. To assess the intra-day precision, six replicates of each concentration were prepared and subjected to the same development conditions as described previously in Sect. 2.5.

To analyze the inter-day precision, six replicates of each concentration level were prepared and analyzed over three consecutive days. The precision was evaluated using the relative standard deviation (RSD%), while accuracy was determined by calculating the mean percentage recovery.

#### Robustness

The robustness was estimated by measuring the ability of the developed method to remain unaffected by small, intentional variations in some parameters taking the peak area of the band as a measure for the robustness. Those investigated parameters were mobile phase compositions (± 0.1 mL), saturation time (± 2 min), migration distance of the mobile phase (± 2 mm) and the detection wavelength (± 2 nm).

## Results and discussion

### Spectral analysis

**Fig. 2:**
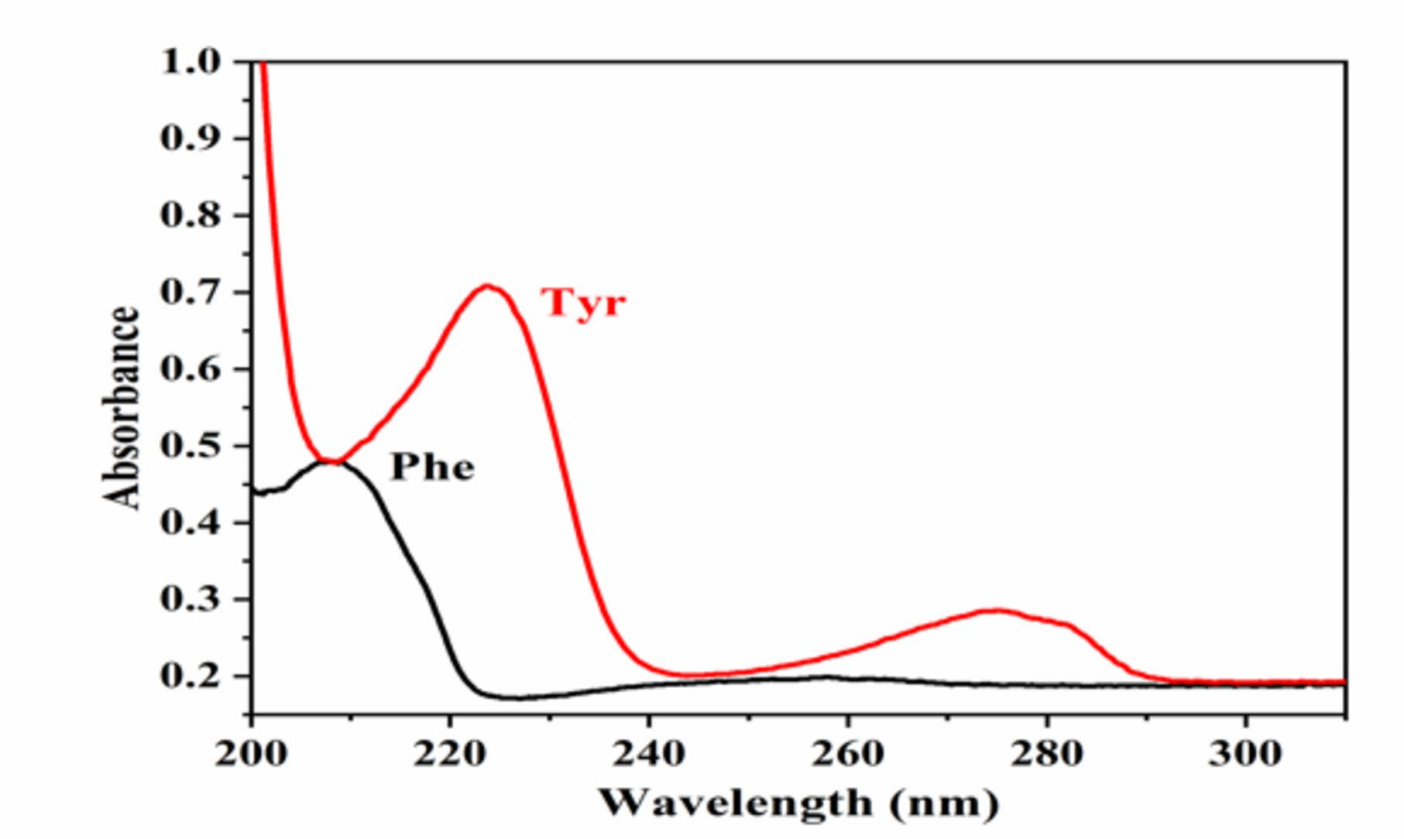
Absorption spectrum of 300 ng band^-1^ for Phe and Tyr.

The absorption spectrum was scanned in the range 200–450 nm for both amino acids (Fig. 2). For Phe, one absorption peak can be detected at 210 nm. Tyr has two absorption peaks can be detected at 225 nm and 280 nm. The highest intensity was recorded at 225 nm; therefore, it was selected for its determination.

### Optimization of the chromatographic conditions

#### Mobile phase composition

Phe and Tyr are highly structurally related compounds, so their chromatographic separation was highly difficult, and several trials were carried out to separate them with acceptable Rf values.

Tyr is more polar than Phe due to the presence of hydroxyl group in the para position, the separation of them is depended on this difference in polarity as Phe was less bounded to silica gel than Tyr. pk_a_ and pk_b_ of carboxylate and amino groups in Phe are 1.83 and 9.13, while in Tyr are 2.20 and 9.11; respectively. Additionally, Tyr has a side chain of hydroxyl group with pk_b_ value of 10.07^17^. This means that a basic mobile phase was required to allow the ionization of hydroxyl group making Tyr more bounded to the silica gel than Phe.

**Fig. 3:**
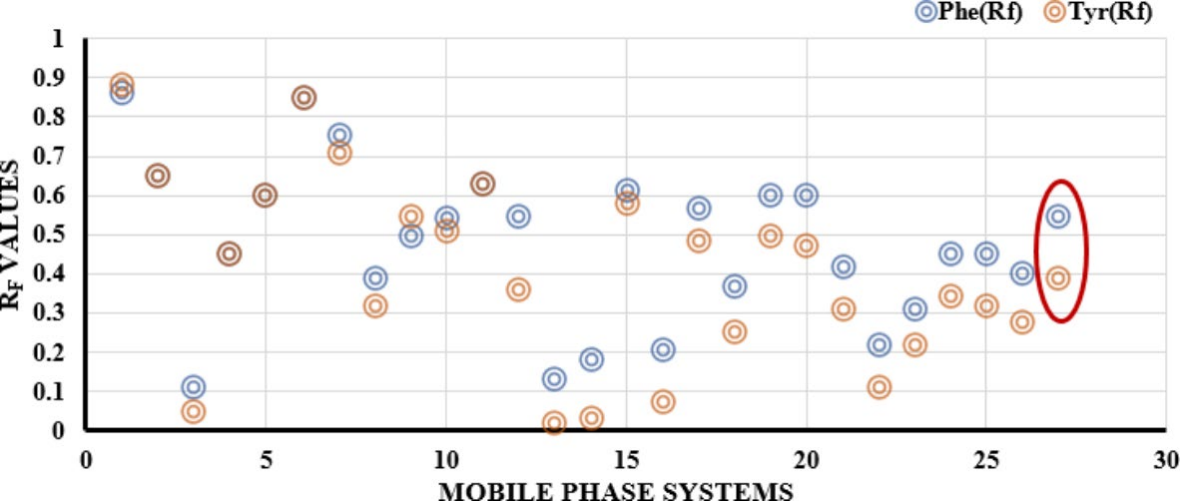
Comparison chart showing the differences between the values of Rf for both Phe and Tyr in the different tested solvent systems

As shown from Tables 1and Fig. 3 several mobile phase systems were tried to separate them. It was observed that the presence of highly non-polar solvents such as chloroform, n-hexane, toluene and 1,2 dichloroethane do not improve the separation efficiency even with the high basicity of the systems using ammonia solution. Moreover, the presence of protic polar solvent only such as methanol, propanol, or ethanol did not enhance the separation efficiency. This indicate that the presence of amphiprotic solvent such as acetonitrile is essential for their separation under the basic conditions. The mobile phase system that consists of acetonitrile, ethyl acetate and ammonia solution could separate both amino acids even with low ammonia concentration. However, due to a miscibility issue between the solvents used, which made a problem during the densitometric determination, we do not use that mobile phase system. Additionally, it was observed that the addition of polar protic solvent enhanced the solvents miscibility with the preservation of its separation efficiency. Different solvents with different ratios were tried such as methanol, ethanol, isopropanol and butanol. The optimum solvent was ethanol.

**Fig. 4:**
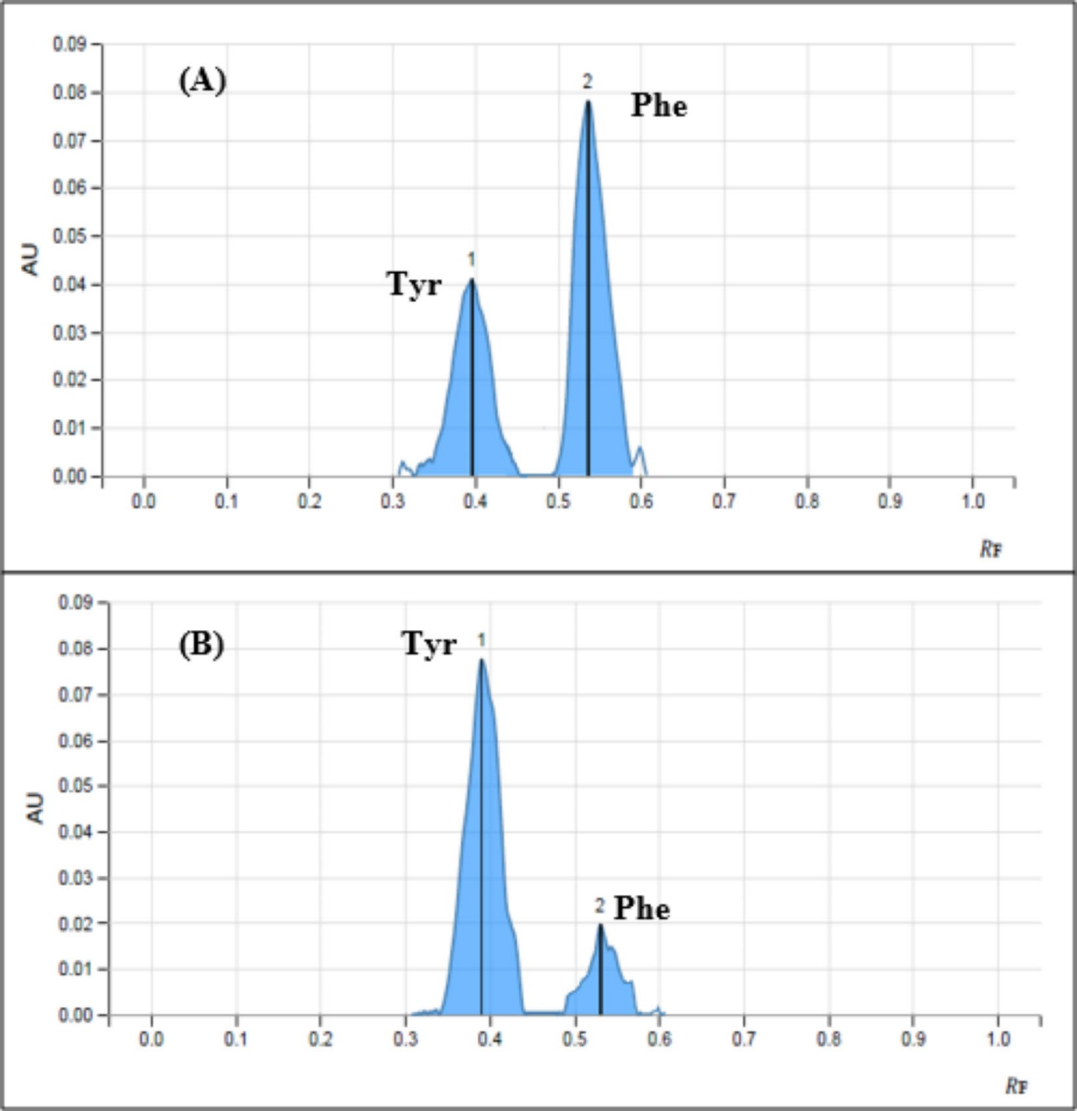
Two-dimensional TLC densitogram of mixture containing 700 ng band^− 1^ of Phe (0.53) and 600 ng bang^− 1^ for Tyr (0.39) using acetonitrile: ethanol: 25% ammonia solution: ethyl acetate (6.5:1.5:1:0.5 v/v/v/v), **(A)** at 210 nm, **(B)** at 225 nm.

On the other hand, the use of ethyl acetate as moderately polar solvent was preferred than the non-polar solvent hexane. Finally, after a careful optimization for the mobile phase compositions, the optimum system was acetonitrile: ethanol: 25% ammonia solution: ethyl acetate (6.5:1.5:1:0.5 v/v/v/v) with Rf values of 0.53 for Phe and 0.39 for Tyr as shown in Fig. 4.

**Table 1:** Trials that was performed for the separation of phe and tyr.

System	Mobile phase composition	Ratio (by volume )	Rf values^*^	
			Phe	Tyr
1	Chloroform: methanol: 17% ammonia solution	2:2:1	0.86	0.88
2	n-propanol: ethylene glycol: ethyl acetate	5:3:2	0.65	0.65
3	n-hexan: n-butanol: 33% ammonia solution	2:7:3	0.11	0.05
4	Toluene: n-propanol: acetic acid	2:7:1	0.45	0.45
5	n-hexane: n-propanol: acetic acid	1:7:1	0.6	0.6
6	Acetonitrile: water	5:5	0.85	0.85
7	Acetonitrile: water: 33% ammonia solution	5:5:0.5	0.75	0.71
8	Acetonitrile: water: 33% ammonia solution	7:3:0.5	0.39	0.32
9	Acetonitrile: water: 33% ammonia solution: butanol	7:3:1:0.1	0.5	0.55
10	Acetonitrile: 0.1 N ammonium acetate buffer (pH 5.4)	7:2	0.54	0.51
11	Acetonitrile: water: ethyl acetate	7:2:1	0.63	0.63
12	Acetonitrile: ethyl acetate: 3.75% ammonia solution	5.5:2:2	0.55	0.36
13	Acetonitrile: ethyl acetate: 3.75% ammonia solution: toluene	5.5: 2:2:0.5	0.13	0.018
14	Acetonitrile: ethyl acetate: 3.75% ammonia solution: n-hexane	5.5:2:2:0.5	0.18	0.036
15	Ethanol: ethyl acetate: 3.75% ammonia solution: n-hexane	5.5:2:2:0.1	0.61	0.58
16	Acetonitrile: ethyl acetate: 3.75% ammonia solution: 1,2 dichloroethane	5.5:2:2:0.5	0.21	0.07
17	Acetonitrile: methanol: 7% ammonia solution: ethyl acetate	5.5: 1: 1: 0.5	0.57	0.48
18	Acetonitrile: methanol: 25% ammonia solution: ethyl acetate	6.5: 2.5:0.5: 1.5	0.37	0.25
19	Acetonitrile: methanol: 25% ammonia solution: ethyl acetate	6.5:2.5:1:1	0.6	0.5
20	Acetonitrile: methanol: 25% ammonia solution: ethyl acetate	6.5:2.5:1:0.5	0.6	0.47
21	Acetonitrile: isopropanol: 25% ammonia solution: ethyl acetate	6.5:2.5:1:0.5	0.42	0.31
22	Acetonitrile: n-butanol: 25% ammonia solution: ethyl acetate	6.5:2.5:1:0.5	0.22	0.11
23	Acetonitrile: ethanol: 25% ammonia solution: n-hexane	6.5:1.5:1:1	0.31	0.22
24	Acetonitrile: ethanol: 25% ammonia solution: n-hexane	6.5: 1.5: 1: 0.5	0.45	0.34
25	Acetonitrile: ethanol: 25%ammonia solution: ethyl acetate	6.5:2.5:1:0.5	0.45	0.32
26	Acetonitrile: ethanol: 25%ammonia solution: ethyl acetate	6.5:0.5:1:0.5	0.4	0.28
**27**	**Acetonitrile: ethanol: 25%ammonia solution: ethyl acetate**	**6.5:1.5:1:0.5**	**0.53**	**0.39**

^*^n = 3.

#### Migration distance

The distance of migration was adjusted in order to obtain Rf values that were reasonable. Th optimum distance for migration was 80 mm. That distance was reached by the mobile phase within approximately 12 ± 0.1 min.

#### Saturation time

The extent of saturation of the mobile phase vapors was fine-tuned in order to achieve consistent results across all measurements. Various saturation times were investigated ranging from 10 to 30 min. It was determined that the most suitable saturation time was approximately 15 min.

### Validation of the developed method

#### Selectivity

The ability of the developed method to separate both Phe and Tyr, which are closely located, is measured by the separation factor (α). They are separated at different Rf values (0.53 for Phe and 0.39 for Tyr) as shown in Fig. 4 and well separated in serum samples (blank serum sample is presented in Fig. 1S).

This value can be computed using Equ.1 where **Rf**_**1**_ and **Rf**_**2**_ are the retardation factor for Tyr and Phe, respectively. For effective separation, the separation factor (α) should be greater than 1.0. Upon calculation, it was determined to be **1.91**, indicating a successful separation.

Calculating the resolution factor (Rs) is also crucial to confirm the separation selectivity and accuracy of the suggested method for quantitatively analyzing Phe and Tyr. The formula for determining the resolution (Rs) was presented in Equ.2.

In the used equation, **z**_**1**_ and **z**_**2**_ refer to the migration distance of Phe and Tyr, while **w**_**1**_ and **w**_**2**_ represent the width of the two adjacent peaks of Phe and Tyr, respectively.

To verify a good resolution, Rs value should exceed 1.0. Upon computing the resolution factor, it was found to be **2.12**, indicating a satisfactory resolution capability of the chromatographic system.

Additionally, we can determine the broadening of the chromatographic spot and the efficiency of the chromatographic plates by evaluating the number of theoretical plates (N) in the chromatographic system using Equ.3.

Where the values of $\iota$ and **z** correspond to the migration distances of the mobile phase and solutes (Phe and Tyr), respectively, while **w** represents the width of the chromatographic peak. The computed values for N were 1086.42 plates cm^-1^ for Phe and 1369.55 plates cm^-1^ for Tyr, providing an estimate of the chromatographic spot broadening and plate separation efficiency for each solute.

Furthermore, the efficiency of the chromatographic system employed can be determined by computing H or HETP (height equivalent to a theoretical plate) using Equ.1.

The computed values for H were 0.0064 cm plate^-1^ for Phe and 0.0051 cm plate^-1^ for Tyr. High N values and very low HETP values proved excellent separation efficiency.

#### Linearity and range, limit of detection (LOD) and limit of quantitation (LOQ)

Under the optimum experimental conditions, calibration curves were constructed for both amino acids by plotting the peak area against the final concentrations as ng band^-1^. Linearity of the method was determined against seven concentrations of six replicates for each concentration as shown in Fig. 2S. The concentration range, parameters of least square equation, correlation and determination coefficients are cited in Table 2.

**Table 2:** Summary of the linearity parameters of the developed HPTLC method.

Parameter	Phe	Tyr
Detection wavelength(nm)	210	225
Rf	0.53 ± 0.05	0.39 ± 0.05
Working linearity range (ng band^− 1^) or (µg ml^− 1^)	50–700 Or 10–140	50–600 Or 10–120
Correlation coefficient(r)	0.9993	0.9989
Determination coefficient (r^2^)	0.9985	0.9980
Intercept (a) ± SD^*^(×10^− 5^)	84.0 ± 2.84	68.0 ± 1.31
Slope (b) ± SD (×10^− 7^)	65.4 ± 3.19	75.0 ± 2.41
LOD (ng band^− 1^) Or (µg ml^− 1^)	14.34 Or 2.87	5.75 Or 1.15
LOQ (ng band^− 1^) Or (µg ml^**− 1**^)	43.46 Or 8.69	17.42 3.48

^*^SD when n=3 .

#### Precision and accuracy

The precision of the suggested method was assessed at three different concentration levels: low, medium, and high. The concentrations tested were 100, 300, and 500 ng band^-1^ for both amino acids.

Six measurements were taken for each of the three concentration levels of Phe and Tyr. These measurements were taken on the same day and on three consecutive days to assess both intra-day and inter-day precision. The precision was expressed as the percentage of relative standard deviation (%RSD). The %RSD did not exceed 4.36 for all concentration levels, indicating that the method had good precision. The accuracy of the method was determined by calculating the mean percentage recoveries for both Phe and Tyr, as presented in Table 3.

**Table 3:** Intra-day and inter-day precision and accuracy of the developed HPTLC method.

Amino acid	Concentration (ng band^− 1^)	Intra-day (*n* = 6)		Inter-day (*n* = 18)	
		% Recovery ± SD	%RSD	% Recovery ± SD^*^	%RSD
Phe	100	98.52 ± 0.72	1.44	98.85 ± 1.14	3.68
	300	98.88 ± 70	0.82	99.34 ± 0.52	3.32
	500	98.97 ± 0.33	0.98	99.41 ± 0.29	4.25
Tyr	100	99.21 ± 0.13	2.81	98.93 ± 0.38	2.99
	300	98.46 ± 0.34	1.16	99.32 ± 0.64	4.32
	500	99.51 ± 0.35	2.85	99.52 ± 0.38	4.36

#### Robustness

Several key parameters were investigated and adjusted slightly to assess the robustness of the suggested method in withstanding minor variations. This was done to determine the reliability of the method under normal operating conditions.

To evaluate the robustness of our method, both Phe and Tyr were tested at a concentration of 300 ng band^-1^. The impact of minor variations in key parameters such as detection wavelength (± 2 nm), mobile phase composition (± 0.1 mL), saturation time (± 2 min), and migration distance (± 2 mm) were examined. The results were expressed as % Recovery ± SD and are presented in Table 4.

**Table 4:** Robustness of the developed HPTLC method at conc. 300 ng band^− 1^ for both phe and Tyr.

Investigated parameters	Phe (% Recovery)		Tyr (% Recovery)	
No variation^*^	99.14 ± 0.51		99.18 ± 0.60	
Detection wavelength	208 nm	97.7 ± 3.22	223 nm	100.86 ± 1.52
	212 nm	98.82 ± 2.49	227 nm	101.32 ± 0.78
Mobile phase compositions (Acetonitrile: ethanol: 25% ammonia: ethyl acetate) 6.4:1.6:1:0.4 6.6:1.4:1:0.6	100.77 ± 1.25 99.84 ± 2.54		101.93 ± 1.81 98.62 ± 3.01	
Migration distance 78 mm 82 mm	100.93 ± 1.21 99.69 ± 2.63		98.69 ± 2.59 99.75 ± 1.01	
Saturation time 13 min. 17 min.	99.31 ± 3.31 99.13 ± 1.25		100.36 ± 0.93 100.19 ± 0.59	

^*^ No variation means using all the optimum conditions.

(Detection wavelength: 210 and 225 nm for Phe and Tyr respectively, migration distance = 80 mm and saturation time 15 min)

### Application to human serum samples

As the kidney is considered the main source for the net release of Tyr to the circulation, so the impairment in kidney functions as in CKD affect the generation of circulating Tyr. The activity of the renal phenylalanine hydroxylase enzyme is reduced in CKD patients so the ratio of Tyr to Phe could be used as a potential biomarker for the early detection of kidney diseases.

Numerous prior studies have indicated that protein precipitation is an effective technique for chromatographic measurement of amino acids in plasma or serum samples^18–20^.

Various precipitating agents, such as methanol, acetonitrile, and perchloric acid, were examined. Perchloric acid yielded the most favorable densitogram for both amino acids. These findings align with the earlier research conducted by Sedgwich and colleagues, who extensively investigated the impact of diverse protein precipitating agents on the retrieval of various amino acids, including the amino acids Phe and Tyr that we focused on in our study^21^.

The effects of various concentrations of perchloric acid were investigated. Concentrations below 5% were insufficient to fully precipitate serum proteins, while concentrations higher than 5% caused damage to the silica material. Different ratios of serum to 5% perchloric acid were tested, specifically 2:1, 1:1, and 1:2. It was observed that the most favourable densitograms, characterized by excellent peak resolution and high recovery, were obtained with the 2:1 ratio. Increasing the perchloric acid ratio beyond this ratio proved harmful to the stationary phase, negatively impacting the recovery of both amino acids and the sharpness of the peaks.

Further optimization was performed by the testing of 5% HClO_4_ in water, acetonitrile, and methanol. The highest recovery was observed when utilizing 5% HClO_4_ in methanol. The use of methanol had an additional benefit in reducing the analysis time for samples.

To enhance the retrieval of both amino acids, the acidity of the supernatant was diminished by introducing a small quantity of 0.2 mol L^-1^ NaOH. The addition of 50 µL of NaOH proved to be the most effective and consistent approach, yielding the best and most reproducible results.

Healthy individuals and CKD patients’ serum samples were treated with the previous mentioned procedure for the determination of both amino acids in Sect. 2.4.

**Fig. 5:**
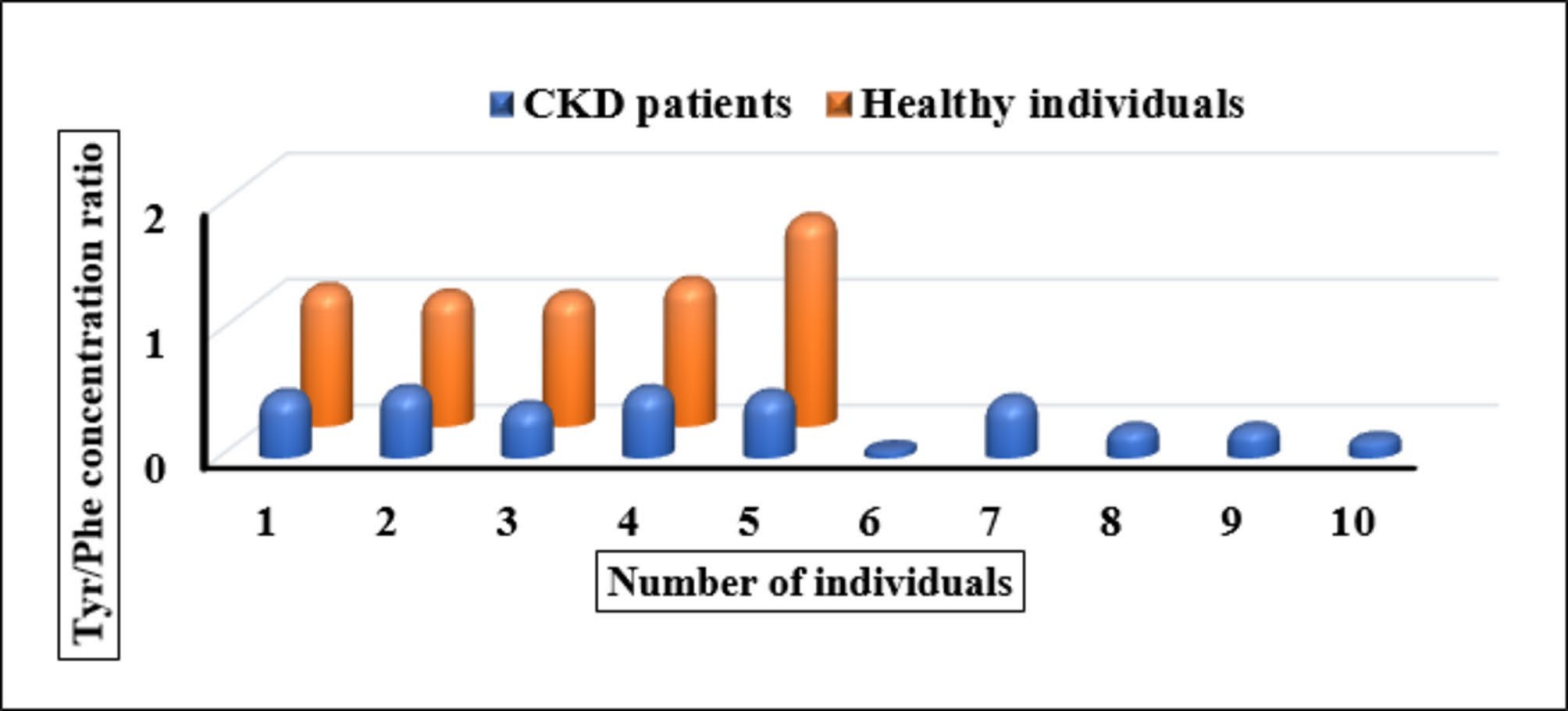
Bar graph for the Tyr/Phe ratio in the serum samples of both healthy individuals and CKD patients.

There was a significant difference in the values of Tyr/Phe concentration ration in both investigated groups as shown in Fig. 5.

The data was analysed by ANOVA test and the statistical comparison between the two groups was estimated using *F*-test and t- test. The obtained statistical data showed that there is a significant difference in the Tyr/Phe concentration ratio between the two investigated groups with P- value < 0.05. Moreover, the calculated t and *F* values were higher than their critical value which implies that the difference in Tyr/Phe concentration ratio is significant as shown in Table 5.

**Table 5:** The statistical comparison for healthy individuals (*n* = 5) and CKD patients (*n* = 10) serum levels for Tyr/Phe ratio.

Statistical value	CKD patients (*n* = 10), healthy individuals (*n* = 5)
mean ± SD	0.36 ± 0.17 for CKD patients, 1.19 ± 0.26 for healthy individuals
P – value	4.90 × 10^− 6^
t – test	t_calculated_ = 6.47, t_critical_ = 1.94
*F* – test	*F*_calculated_ = 55.35, *F*_critical_ = 4.66

### Greenness assessment

Analysts face a significant obstacle in creation of an environmentally friendly analytical method. It involves not only meeting analytical performance standards such as selectivity, specificity, and limit of detection, but also ensuring that the method is environmentally sustainable. This challenge arises from the requirement to strike a balance between environmental and analytical concerns during the method development process. To assess the degree of environmental sustainability achieved, two different tools were used: the Analytical Greenness Metric (AGREE) and the Green Analytical Procedure Index (GAPI).

AGREE provides a thorough, flexible, and simple evaluation procedure that yields results that are clear and instructive. The criteria used by AGREE are based on the 12 GAC (green analytical chemistry) principles and are then scaled from 0.0 to 1.0^22^. The plot shows different coloured zones (light green, dark green, red), which suggests that different weights were used for the principles in the AGREE greenness assessment. The weights assigned to each principle in the AGREE method can impact the overall greenness assessment. Light green zones indicate a moderate or acceptable level of greenness, dark green zones indicate a higher level of greenness, while red or orange zones represent processes that have a lower level of greenness.

**Fig. 6:**
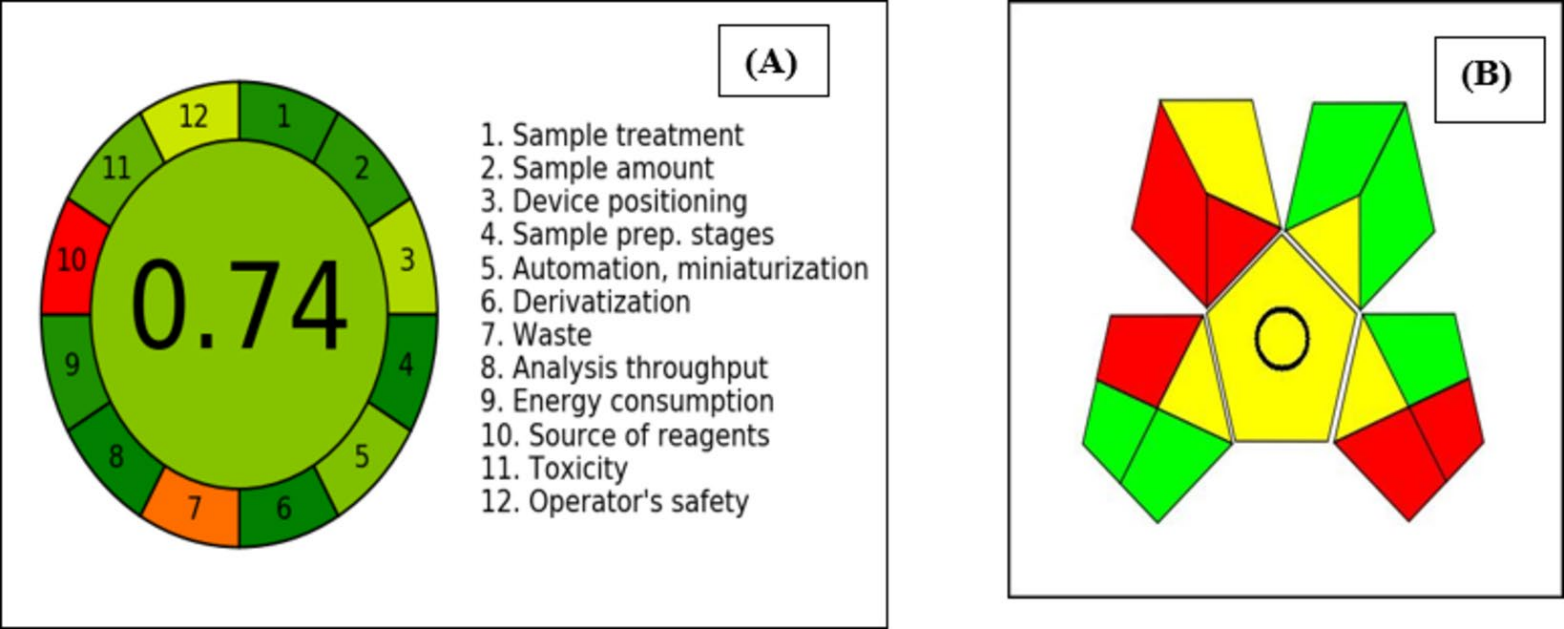
AGREE result (**A**) and GAPI pentagram (**B**) of the developed HPTLC method.

The obtained score of 0.74, shown in Fig. 6A, indicates the developed strategy’s remarkable environmental friendliness. Any number below 0.50 denotes that the analytical method is unsatisfactory, while an eco-scale value of 0.50 or greater is regarded as acceptable for drug analysis^23,24^. The only two red regions were account for waste amounts and the source of used reagents as all of them not from bio-based source.

GAPI is a recently developed tool for gauging the ecological impact of analytical techniques. It evaluates the environmental friendliness of the entire analytical procedure, starting with the collection of samples and culminating in the final determination. The tool is composed of five pentagrams, each of which corresponds to a distinct phase of the analytical process. Each stage is color-coded to represent one of three levels of environmental impact: green for minimal impact, yellow for moderate impact, and red for high impact^25^. Figure 6B shows that only five red zones were observed, indicating that the method being developed is green. The first red zone was accounts for the using of special storage conditions for the collected samples as freezing of serum samples is required to avoid their damage. The second was due to the use of protein precipitation technique for extraction, and this considered as macroextraction procedure, but this extraction method was reported as the most efficient in the determination of amino acids in biological fluids. To reduce that environmental risk, we reduced the amount of precipitating agent to just 150 µL. The third red zone was due to the use of ammonia solution as non-green solvent although the rest used solvent in the mobile phase are considered green. The use of ammonia solution was essential for their separation that it was not avoidable. The reason for the fourth red zone was that the amount of waste produced exceeded 10 mL, but a significant amount of mobile phase was necessary to properly saturate the development tank. As for the final red region, it was due to a lack of waste management procedures in place. According to the eco-scale value obtained in this study, the proposed method is considered an outstanding green analytical technique for measuring Phe and Tyr.

### Comparison of the proposed method with other reported methods

The proposed high-performance thin layer chromatography (HPTLC) method for the simultaneous determination of tyrosine and phenylalanine in serum offers distinct advantages when compared to other established techniques such as spectrophotometry, high-performance liquid chromatography (HPLC), and voltammetric methods as indicated in Table 6. Spectrophotometric techniques, while straightforward and cost-effective, often lack the sensitivity and specificity required for individual amino acid detection in complex biological matrices. HPLC, on the other hand, is highly sensitive and capable of detailed quantitative analysis but typically involves intricate sample preparation and high operational costs. Voltammetric methods can provide rapid and sensitive measurements but may require specific electrode modifications and careful optimization of experimental conditions. In contrast, the HPTLC method combines ease of use with the ability to analyse multiple samples simultaneously, potentially lowering costs and reducing analysis time. However, its sensitivity may not match that of HPLC, highlighting the need for thorough validation to establish its efficacy and reliability in clinical applications. Overall, while each method has its strengths, the HPTLC approach could serve as a complementary technique, particularly in resource-limited settings.

**Table 6:** Performance comparison of different methods for the determination of Phe and/or tyr.

Amino acid (s)	Technique	LOD	Ref
Phe	Spectrophotometry	31.6 µmol l^− 1^	^26^
Tyr andTrp	HPLC-FLD	0.014 µmol l^− 1^ for Tyr	^27^
Phe and Tyr	HPLC-FLD	0.3 µmol l^− 1^ for Phe and Tyr	^28^
Trp and Tyr with their metabolites	HPLC-ESI-MS	60.0 nmol l^− 1^ for Tyr	^29^
Tyr	Differential pulse voltammetry	2.7 nmol l^− 1^	^30^
Phe	Differential pulse voltammetry	47.0 nmol l^− 1^	^31^
Phe and Tyr	HPTLC	17.37 µmol l^− 1^ for Phe and 6.35 µmol l^− 1^ for Tyr.	This work

## Conclusion

A novel, sensitive, and straightforward HPTLC densitometric approach was created to measure the levels of Phe and Tyr in the serum of both healthy individuals and CKD patients using the standard addition method. The results obtained showed that there is a significant difference in Tyr/Phe concentration ratio between the two groups under investigation. Therefore, Tyr/Phe ratio could be considered as a measure for phenylalanine hydroxylase enzyme activity in CKD patients which making this ratio as a potential biomarker for the detection of the disease in its early stages. The validation parameters for the new method were deemed satisfactory. The values for separation and selectivity factors, theoretical plates, and height equivalents to a theoretical plate confirmed the efficiency of the chromatographic system used and the accuracy of quantitative analysis. The method has the added benefits of analysis time reduction and cost-effectiveness, and multiple samples can be analyzed simultaneously. Additionally, the greenness of the method was verified through AGREE and Complex GAPI tools indicating that the excellent greenness properties of the developed method.

## Electronic supplementary material

Below is the link to the electronic supplementary material.

Supplementary Material 1

## Data Availability

The authors declare that the data supporting the findings of this study are available within the paper. Should any raw data files be needed in another format they are available from the corresponding author upon reasonable request. All data are provided within the paper.
