# Supplementary material for: A sustainable HPTLC approach for green assessment of Tyrosine to phenylalanine ratio in chronic kidney disease
Source: Sci Rep. 2024 Nov 17;14:28371. doi: 10.1038/s41598-024-79611-9 (PMC11570637; doi:10.1038/s41598-024-79611-9)
Supplement: Supplementary file 1 — Supplementary Material 1 [file 41598_2024_79611_MOESM1_ESM.docx]

**A sustainable HPTLC Approach for Green Assessment of Tyrosine to Phenylalanine Ratio in Chronic Kidney Disease**

**Rania M. Kamel^a^, Fatma A. M. Abdel-aal^a,*^,** **Mostafa M. Osman^b^, Fardous A. Mohamed^a^, Abdel-Maaboud I. Mohamed^a^**

**^a^ Pharmaceutical Analytical Chemistry Department, Faculty of Pharmacy, Assiut University, 71526 Assiut, Egypt.**

**^b^ Urology Department, Faculty of Medicine, Al-Azhar University, Assiut, Egypt.**

*** Corresponding author**

**E-mail address: Fatmamoustafa@aun.edu.eg**

**Tel: +20 882 411009**

**Fax: +20 882 345631**

**Keywords:**

**Biomarkers; Chronic kidney disease; HPTLC; Phenylalanine; Serum samples; Tyrosine**

**
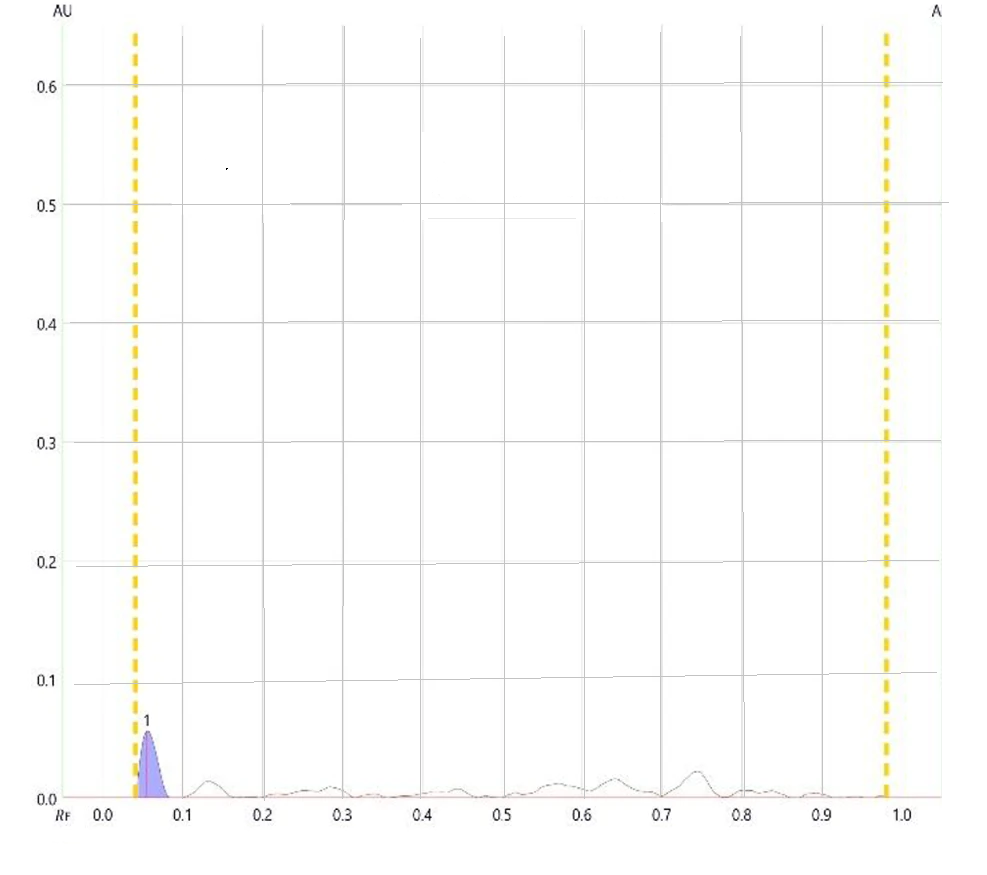
**

**Fig. 1S: Two-dimensional TLC densitograms displays blank serum sample**


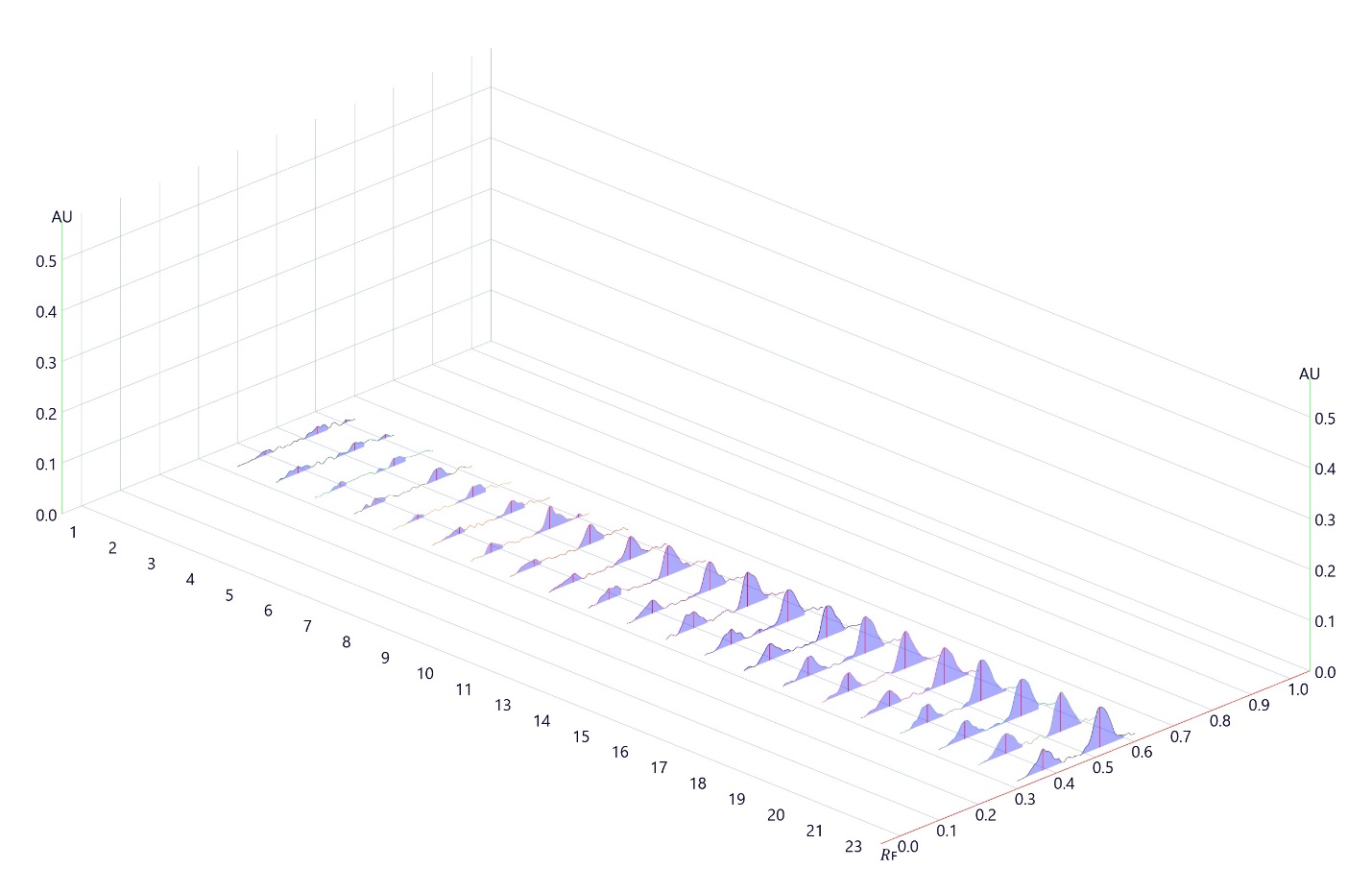


**Fig. 2S: Three-dimensional TLC densitogram of mixture containing different concentrations of (50-700 ng band^-1^) of Phe (0.53) and (50-600 ng bang^-1^) for Tyr (0.39) using acetonitrile: ethanol: 25% ammonia solution: ethyl acetate (6.5:1.5:1:0.5 v/v/v/v) at 210 nm.**
